# Access to Care, Cost of Care, and Satisfaction With Care Among Adults With Private and Public Health Insurance in the US

**DOI:** 10.1001/jamanetworkopen.2021.10275

**Published:** 2021-06-01

**Authors:** Charlie M. Wray, Meena Khare, Salomeh Keyhani

**Affiliations:** 1 Department of Medicine, University of California, San Francisco; 2 Division of Hospital Medicine, San Francisco Veterans Affairs Medical Center, San Francisco, California; 3 Northern California Institute for Research and Education, San Francisco Veterans Affairs Medical Center, San Francisco; 4 Division of General Internal Medicine, San Francisco Veterans Affairs Medical Center, San Francisco, California

## Abstract

**Importance:**

Contemporary data directly comparing experiences between individuals with public and private health insurance among the 5 major forms of coverage in the US are limited.

**Objective:**

To compare individual experiences related to access to care, costs of care, and reported satisfaction with care among the 5 major forms of health insurance coverage in the US.

**Design, Setting, and Participants:**

This survey study used data from the 2016-2018 Behavioral Risk Factor Surveillance System on 149 290 individuals residing in 17 states and the District of Columbia, representing the experiences of more than 61 million US adults.

**Exposure:**

Private (individually purchased and employer-sponsored coverage) or public health insurance (Medicare, Medicaid, and Veterans Health Administration [VHA] or military coverage).

**Main Outcomes and Measures:**

A pairwise multivariable analysis was performed, controlling for underlying health status of US adults covered by private and public health insurance plans, and responses to survey questions on access to care, costs of care, and reported satisfaction with care were compared. Estimates are weighted.

**Results:**

A total of 149 290 individuals responded to the survey (mean [SD] age, 50.7 [0.2] years; 52.8% female). Among the respondents, most were covered by private insurance (95 396 [63.9%]), followed by Medicare (35 531 [23.8%]), Medicaid (13 286 [8.9%]), and VHA or military (5074 [3.4%]) coverage. Among those with private insurance, most (117 939 [79.0%]) had employer-sponsored coverage. Compared with those covered by Medicare, individuals with employer-sponsored insurance were less likely to report having a personal physician (odds ratio [OR], 0.52; 95% CI, 0.48-0.57) and were more likely to report instability in insurance coverage (OR, 1.54; 95% CI, 1.30-1.83), difficulty seeing a physician because of costs (OR, 2.00; 95% CI, 1.77-2.27), not taking medication because of costs (OR, 1.44; 95% CI, 1.27-1.62), and having medical debt (OR, 2.92; 95% CI, 2.69-3.17). Compared with those covered by Medicare, individuals with employer-sponsored insurance were less satisfied with their care (OR, 0.60; 95% CI, 0.56-0.64). Compared with individuals covered by Medicaid, those with employer-sponsored insurance were more likely to report having medical debt (OR, 2.06; 95% CI, 1.83-2.32) and were less likely to report difficulty seeing a physician because of costs (OR, 0.83; 95% CI, 0.73-0.95) and not taking medications because of costs (OR, 0.78; 95% CI, 0.66-0.92). No difference in satisfaction with care (OR, 0.96; 95% CI, 0.87-1.06) was found between individuals with employer-sponsored private health insurance and those with Medicaid coverage.

**Conclusions and Relevance:**

In this survey study, individuals with private insurance were more likely to report poor access to care, higher costs of care, and less satisfaction with care compared with individuals covered by publicly sponsored insurance programs. These findings suggest that public health insurance options may provide more cost-effective care than private options.

## Key Points

**Question:**

What are individuals’ experiences with access to care, costs of care, and satisfaction with care among the 5 major forms of health insurance coverage in the US?

**Findings:**

In this survey study of 149 290 individuals residing in 17 states and the District of Columbia, individuals with employer-sponsored and individually purchased private insurance were more likely to report poor access to health care, higher costs of care, and less satisfaction with care compared with individuals covered by publicly sponsored insurance programs.

**Meaning:**

The findings suggest that efforts to increase the number of individuals covered by public insurance programs or improve protections for individuals covered by private insurance against increasing costs are needed.

## Teaser

This survey study compares individual experiences related to access to care, costs of care, and reported satisfaction with care for the 5 major forms of health insurance coverage in the US.

## Introduction

In the past decade, health insurance expansion has been a major aspect of health care reform in the US, with the Patient Protection and Affordable Care Act (ACA) increasing coverage to more than 20 million US adults. The ACA has expanded access to care and has been associated with improvements in self-reported health and reductions in mortality among previously uninsured US adults.^1,2,3,4,5,6,7,8,9,10,11^

As a result of the ACA, the number of both Medicaid and individually purchased private insurance coverage options has increased, whereas the number of employer-sponsored insurance options has decreased.^12^ In recent years, policy makers have called for legislation that would further expand coverage, with proposals including developing a public option, reducing the Medicare eligibility age to 50 years, and expanding Medicare coverage to all US adults. Policy makers have also proposed reducing out-of-pocket costs by increasing subsidies to plans offered on state health insurance exchanges and by expanding eligibility parameters for health insurance subsidies.^13^

Research from more than a decade ago suggests that Medicare enrollees were more likely to rate their insurance positively compared with those enrolled in private plans and that newly insured US adults with Medicare reported more satisfaction with care compared with US adults not yet covered by the program.^14,15^ Prior research has also demonstrated that out-of-pocket spending increased more rapidly among individuals covered by employer-sponsored insurance and decreased among individuals covered by Medicare.^16^ In addition, Medicaid has been compared with private insurance; however, the research is limited to certain populations^17,18,19^ or receipt of specific services^20,21,22^ and has shown mixed results. To our knowledge, no contemporary data directly comparing the experiences between US adults with public and private health insurance among the 5 major forms of coverage have been published.

We used the Behavioral Risk Factor Surveillance System (BRFSS)^23^ to compare experiences related to access to care, costs of care, and satisfaction with care among the 5 major forms of health insurance coverage (private employer–sponsored insurance, private individually purchased insurance, Medicare, Medicaid, and Veterans Health Administration [VHA] or military coverage) after accounting for respondents’ underlying health.

## Methods

### Data Source

This survey study used data from the BRFSS, a state-based telephone survey used for the annual collection of data from a representative sample of noninstitutionalized US adults, aged 18 or older, from January 1, 2016, to December 31, 2018. During this period, the BRFSS survey included 6 questions related to the domains of access to care, costs of care, and satisfaction with care. A detailed description of the BRFSS survey design, questionnaires, and data collection is available on the BRFSS website.^23^ This study was deemed to be exempt from institutional review board approval by the San Francisco Veterans Affairs Medical Center institutional review board, with a waiver of informed consent, because it used publicly available data. This study followed the American Association for Public Opinion Research (AAPOR) reporting guideline.

### Analytic Sample

The BRFSS survey included a health care access module that was administered to a random sample of adults in 17 states and the District of Columbia. Our analysis included all US adults who responded to the 2016-2018 health care access module. To assess the type of coverage, we used the question, “What is the primary source of your health care coverage?” The response options included the following: (1) plan purchased through an employer or union; (2) plan that you or another family member buys on your own; (3) Medicare; (4) Medicaid or other state program; (5) Tricare, VHA, or military; (6) Alaska Native Health System, Indian Health Service, or Tribal Health Services; (7) other source of coverage; and (8) no coverage. If the respondents indicated that they purchased insurance through the Health Insurance Marketplace, they were then asked whether it was a private plan purchased on their own or by a family member or whether they received Medicaid (public plan). A total of 169 892 adults were interviewed with this question, with a response rate of 67%. We excluded 15 254 individuals who were uninsured; 768 individuals covered by the Alaska Native Health System, Indian Health Service, or Tribal Health Services; and 4580 individuals who reported some other form of coverage. These exclusions resulted in an analytic sample of 149 290 adults representing 61 million US adults from 17 states and the District of Columbia.

### Independent Variables and Covariates

We divided the 149 290 respondents to 5 categories: those with a public form of insurance (Medicare, Medicaid, or VHA or military) and those with a private form of insurance (employer sponsored or individually purchased). We extracted data on sex, race/ethnicity, marital status, educational attainment, employment status, and annual household income. To account for baseline differences in health, we used the following question on comorbidity: “Has a health professional ever told you that you had any of the following?” The conditions that were common to all years included obesity, coronary artery disease, chronic obstructive pulmonary disease, stroke, diabetes, kidney disease, and cancer. We categorized individuals based on the reported number of comorbidities (none, 1, 2, or >2). Functional impairment was assessed using 4 questions: (1) “Because of a physical, mental, or emotional condition, do you have serious difficulty concentrating, remembering, or making decisions?” (2) “Do you have serious difficulty walking or climbing stairs?” (3) “Do you have difficulty dressing or bathing?” (4) “Because of a physical, mental, or emotional condition, do you have difficulty doing errands alone such as visiting a physician’s office or shopping?” We categorized individuals based on the number of impairments they reported (none, 1, or ≥2). Missing data ranged from 0% to 2.5%.

### Outcomes

We examined 3 domains of care among this insured sample: (1) experiences related to access to care, (2) costs of care, and (3) satisfaction with care. Experience with access to care was assessed with the following 2 questions: (1) “Do you have one person you think of as your personal physician or health care provider?” (2) “In the past 12 months is there any time when you did not have any health insurance?” Experience with costs of care was assessed by the following 3 questions: (1) “Was there a time in the past 12 months when you needed to see a physician but could not because of cost?” (2) “In the past 12 months was there a time when you did not take your prescription medications due to cost?” (3) “Do you currently have any health care bills that are being paid over time?”

Satisfaction with care was assessed with the question, “In general, how satisfied are you with the care you received?” Responses were dichotomized as very satisfied and somewhat satisfied or not at all satisfied. Missing data ranged from 0.2% to 1.8%.

### Statistical Analysis

Weighted estimates were calculated by taking the survey stratum and state-based sampling weights into account. First, we calculated descriptive statistics and examined the sociodemographic and health characteristics of individuals covered by different forms of health insurance. Second, we examined differences related to access to care, costs of care, and satisfaction among those covered by public and private health insurance. Third, we conducted a pairwise multivariable logistic regression comparing experiences related to access to care, costs of care, and satisfaction with care among individuals covered by each private plan (individually purchased and employer-sponsored coverage) compared with those covered by each public plan (Medicare, Medicaid, and VHA or military coverage) after accounting for health status, which studies have shown can affect experiences and satisfaction with care.^24,25^ We did not adjust for sociodemographic characteristics because age, race/ethnicity, sex, and income are correlated with coverage programs in the US. For example, Medicare primarily covers individuals 65 years or older; Medicaid primarily covers low-income populations; private insurance primarily covers younger, employed individuals; and VHA and military insurance primarily covers men. Individuals with missing data (6.4% to 7.9%) were excluded from analysis. Statistical analyses were performed using SAS statistical software, version 9.4 (SAS Institute Inc). Statistical significance was defined as *P* < .05.

## Results

### Baseline Characteristics

A total of 149 290 individuals responded to the survey (mean [SD] age, 50.7 [0.2] years; 52.8% female). Among the 149 290 respondents who reported having health insurance, most were covered by private insurance (95 396 [63.9%]), followed by Medicare (35 531 [23.8%]), Medicaid (13 286 [8.9%]), and VHA or military insurance (5074 [3.4%]). Among those with private insurance, most (117 939 [79.0%]) had employer-sponsored coverage. Sociodemographic characteristics of individuals covered by public and private insurance varied widely. Overall, younger, employed, and married individuals as well as those with higher educational attainment were covered with private insurance; those with public plans were older, unemployed, and had lower educational attainment (Table 1).

**Table 1.** Baseline Comparison of Sociodemographic Characteristics Among US Adults With Different Types of Health Care Coverage^a^

Characteristic	Insured US adults	Adults with private insurance		Adults with public insurance		
		Employer sponsored	Individually purchased	Medicare	Medicaid	VHA or military
Respondents, No. (weighted %)	149 290 (100)	65 312 (50.4)	17 286 (13.5)	48 812 (23.8)	12 270 (8.9)	5610 (3.4)
Age, mean (SD), y	50.7 (0.2)	44.4 (0.2)	50.4 (0.5)	69.1 (0.1)	38.2 (0.5)	53.2 (0.9)
Female sex	85 541 (52.8)	35 749 (50.1)	10 024 (53.3)	29 713 (56.4)	8267 (66.9)	1788 (29.6)
Race/ethnicity						
White	114 145 (71.8)	50 559 (73.4)	13 876 (71.9)	38 766 (74.9)	6725 (54.7)	4219 (71.2)
African American	16 982 (13.4)	6799 (11.9)	1347 (10.9)	5474 (13.6)	2711 (23.6)	651 (16.7)
Hispanic	8954 (9.6)	3968 (9.0)	1032 (12.2)	2027 (7.8)	1619 (15.0)	308 (6.2)
Asian, Pacific Islander	1894 (2.8)	1189 (3.7)	280 (3.2)	250 (1.3)	123 (2.0)	52 (1.9)
Other	4718 (2.4)	1744 (2.1)	431 (1.8)	1416 (2.4)	862 (4.7)	265 (3.9)
Married	79 201 (54.2)	42 022 (63.2)	8941 (48.8)	21 866 (48.1)	3234 (28.1)	3138 (55.0)
Educational attainment						
Less than high school	9975 (10.2)	1789 (5.0)	982 (9.7)	4841 (17.4)	2153 (22.9)	210 (6.1)
High school graduate	41 221 (30.2)	14 318 (26.2)	5256 (33.4)	15 437 (34.1)	4678 (38.0)	1532 (29.7)
Some college	41 146 (31.1)	17 637 (32.1)	5039 (31.4)	12 948 (28.3)	23 529 (9.8)	1993 (40.2)
Completed college	56 522 (28.4)	31 397 (36.7)	5956 (25.5)	15 441 (20.3)	1870 (9.3)	1858 (24.0)
Unemployed	74 785 (43.7)	13 444 (19.0)	8670 (47.9)	41 527 (85.4)	7690 (61.9)	3454 (55.6)
Annual household income, $						
<10 000	5370 (4.2)	376 (0.8)	414 (3.5)	2307 (6.8)	2158 (20.0)	115 (2.9)
10 000-19 999	15 319 (11.3)	1495 (3.0)	1693 (12.4)	8191 (22.3)	3411 (32.3)	529 (10.2)
20 000-34 999	24 736 (19.6)	6632 (12.3)	3503 (24.7)	10 609 (29.0)	2883 (30.6)	1109 (22.2)
35 000-49 999	18 157 (14.0)	7985 (13.7)	2523 (17.4)	5974 (14.0)	829 (8.9)	846 (17.8)
50 000-74 999	20 627 (15.9)	11 728 (20.0)	2312 (14.7)	5255 (11.4)	425 (4.0)	907 (18.5)
≥75 000	42 211 (35.0)	29 549 (50.2)	3815 (27.4)	7132 (16.5)	405 (4.2)	1310 (28.4)
General health						
Excellent	24 783 (17.8)	13 647 (21.2)	3433 (21.3)	5562 (11.0)	1285 (11.8)	856 (18.1)
Very good	49 380 (33.1)	25 755 (38.5)	5960 (33.7)	13 427 (25.3)	2634 (23.1)	1604 (30.2)
Good	46 237 (31.0)	19 554 (30.8)	5194 (29.9)	15 684 (32.3)	3982 (31.5)	1823 (30.3)
Fair	20 339 (12.9)	5122 (7.7)	2010 (11.4)	9429 (21.0)	2847 (22.8)	931 (14.9)
Poor	8223 (5.1)	1161 (1.9)	658 (3.7)	4552 (10.6)	1474 (10.8)	378 (6.6)
Comorbidities						
Obesity (BMI >30)	44 588 (31.6)	19 455 (31.5)	4370 (25.9)	14 497 (32.3)	4500 (38.7)	1766 (31.4)
Coronary artery disease	9423 (5.0)	1847 (2.2)	847 (3.8)	5528 (11.3)	716 (5.4)	485 (6.6)
COPD	13 716 (7.8)	2614 (3.8)	1185 (5.6)	7310 (14.9)	1877 (13.7)	730 (11.8)
Stroke	6860 (3.7)	1145 (1.4)	641 (3.1)	3988 (8.5)	744 (4.7)	342 (5.1)
Diabetes	21 580 (12.0)	5840 (7.5)	2017 (9.6)	10 700 (22.0)	2014 (13.8)	1009 (14.2)
Kidney disease	5823 (3.1)	1230 (1.5)	552 (2.7)	3277 (6.4)	522 (3.9)	242 (3.8)
Cancer	26 822 (13.7)	7312 (8.6)	2854 (13.1)	14 108 (26.1)	1441 (9.6)	1107 (14.8)
Functional status						
Difficulty in concentrating or remembering	15 663 (11.3)	3498 (6.1)	1460 (9.7)	6790 (16.9)	3106 (26.5)	809 (15.4)
Difficulty in walking or climbing stairs	27 148 (16.0)	4838 (6.9)	2474 (12.4)	15 025 (33.0)	3539 (25.8)	1272 (21.7)
Difficulty in dressing or bathing	6719 (4.3)	993 (1.6)	507 (2.5)	3633 (8.5)	1266 (9.9)	320 (6.2)
Difficulty in doing errands alone	11 426 (7.4)	1699 (2.7)	934 (5.7)	6167 (14.5)	2131 (17.4)	495 (9.1)
Visits for health care in past 12 mo						
0	11 255 (9.2)	6104 (10.7)	1767 (11.5)	2124 (4.8)	941 (9.1)	319 (6.8)
1-2	47 477 (34.5)	24 360 (38.8)	6113 (36.3)	12 326 (26.6)	2975 (26.5)	1703 (34.9)
>2	84 927 (56.4)	33 691 (50.5)	8914 (52.1)	31 389 (68.7)	7556 (64.4)	3377 (58.3)
Comorbid conditions^b^						
0	68 988 (51.7)	36 540 (59.4)	9116 (58.6)	15 802 (33.6)	5176 (47.0)	2354 (49.8)
1	50 222 (31.9)	21 290 (31.2)	5420 (28.2)	17 440 (35.3)	4256 (33.4)	1816 (29.6)
2	20 462 (11.5)	5799 (7.4)	1977 (10.0)	9944 (20.1)	1851 (13.0)	891 (12.6)
>2	9577 (4.9)	1658 (2.0)	763 (3.2)	5620 (11.0)	987 (6.5)	549 (7.9)
Functional impairments^c^						
0	108 784 (76.7)	55 924 (87.9)	13 304 (80.6)	29 135 (58.5)	6675 (59.1)	3746 (70.3)
1	21 438 (13.5)	5510 (8.6)	2251 (11.9)	10 286 (22.3)	2420 (18.6)	971 (16.0)
≥2	15 391 (9.8)	2236 (3.5)	1257 (7.4)	8310 (19.3)	2829 (22.3)	759 (13.7)

Abbreviations: BMI, body mass index (calculated as weight in kilograms divided by height in meters squared); COPD, chronic obstructive pulmonary disease; VHA, Veterans Health Administration.

^a^ Data are presented as weighted percentages of individuals unless otherwise indicated. States covered in this study included Delaware, Florida, Georgia, Kentucky, Louisiana, Maine, Minnesota, Mississippi, Nebraska, New Hampshire, New Jersey, New Mexico, Oklahoma, Oregon, Pennsylvania, Tennessee, Wisconsin, and the District of Columbia.

^b^ Comorbid conditions included the following: obesity, coronary artery disease, COPD, stroke, diabetes, kidney disease, and cancer.

^c^ Functional impairments included the following: difficulty remembering, difficulty walking, difficulty dressing, difficulty doing errands alone.

### Access, Costs, and Satisfaction

Individuals covered by Medicare more commonly reported having a health care provider (91.7%) compared with individuals covered by other programs (employer sponsored, 80.7%; individually purchased, 79.9%; Medicaid, 77.8%; and VHA or military, 78.8%). Differences among the other plans on this metric were small (Figure). Individuals with Medicare were the most satisfied with their care (70.1%), followed by individuals who had VHA or military coverage (68.3%). Reports of medical debt were more common among individuals who had employer-sponsored private insurance coverage (23.4%) and those with individually purchased private insurance (22.3%) than among individuals in the other groups (Medicare, 15.6%; Medicaid, 18.3%; and VHA or military, 11.9%). Individuals with Medicaid most frequently reported difficulty with seeing a physician because of cost (16.8%), followed by those covered by individually purchased insurance (11.8%). Difficulty taking medications because of costs was most common among those covered by Medicaid (14.3%), followed by those with Medicare (9.7%) and individually purchased private insurance (9.6%).

**Figure.**
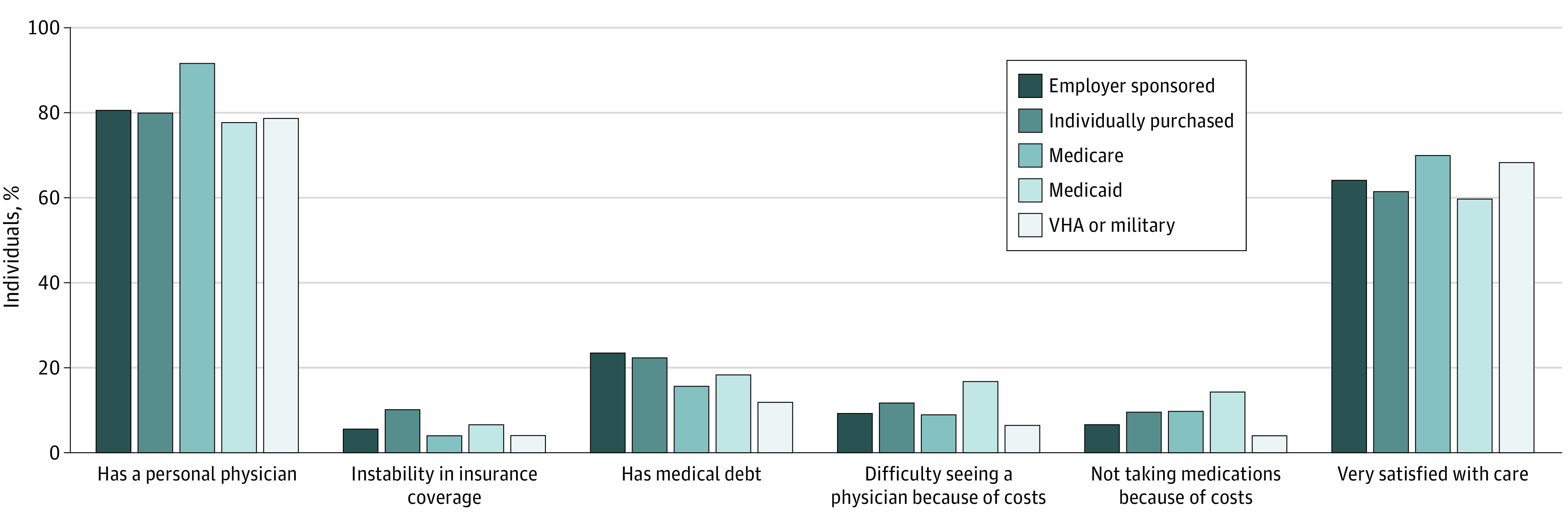
Access to Care, Costs of Care, and Satisfaction With Care Among US Adults With Different Forms of Health Insurance VHA indicates Veterans Health Administration.

### Comparison of Private Insurance and Medicare

In adjusted comparisons of private insurance with Medicare, individuals covered by employer-sponsored insurance were less likely to report having 1 person they considered their personal physician (odds ratio [OR], 0.52; 95% CI, 0.48-0.57) and were more likely to report instability in insurance coverage (OR, 1.54; 95% CI, 1.30-1.83), difficulty in seeing a physician because of costs (OR, 2.00; 95% CI, 1.77-2.27), not taking medication because of costs (OR, 1.44; 95% CI, 1.27-1.62), and having medical debt (OR, 2.92; 95% CI, 2.69-3.17). Overall, individuals covered by employer-sponsored private insurance were less satisfied with their care compared with those covered by Medicare (OR, 0.60; 95% CI, 0.56-0.64) (Table 2 and eTable 1 in the Supplement). Adjusted comparisons between those with individually purchased private insurance and those with Medicare demonstrated similar findings (Table 2 and eTable 2 in the Supplement).

**Table 2.** Pairwise Comparisons of Private Insurance Plans With Public Insurance Plans in Terms of Access to Care, Costs of Care, and Satisfaction With Care After Accounting for Health Status^a^

Insurance type	Health care access		Health care costs			Very satisfied with care
	Has a personal physician	Instability in insurance coverage	Difficulty seeing a physician because of cost	Not taking medications because of costs	Reported medical debt	
**Comparison with private, employer-based coverage**						
Medicare (n = 114 124)						
Unadjusted OR (95% CI)	0.38 (0.35-0.41)	1.38 (1.19-1.60)	1.04 (0.95-1.14)	0.66 (0.60-0.73)	1.65 (1.55-1.76)	0.76 (0.72-0.81)
Adjusted OR (95% CI)	0.52 (0.48-0.57)	1.54 (1.30-1.83)	2.00 (1.77-2.27)	1.44 (1.27-1.62)	2.92 (2.69-3.17)	0.60 (0.56-0.64)
Medicaid (n = 77 582)						
Unadjusted OR (95% CI)	1.19 (1.08-1.32)	0.29 (0.26-0.33)	0.50 (0.45-0.56)	0.43 (0.37-0.49)	1.36 (1.23-1.51)	1.21 (1.11-1.32)
Adjusted OR (95% CI)	1.58 (1.40-1.77)	0.34 (0.29-0.39)	0.83 (0.73-0.95)	0.78 (0.66-0.92)	2.06 (1.83-2.32)	0.96 (0.87-1.06)
VHA or military (n = 70 922)						
Unadjusted OR (95% CI)	1.13 (0.97-1.3)	1.37 (0.97-1.93)	1.48 (1.17-1.86)	1.68 (1.19-2.38)	2.25 (1.90-2.67)	0.83 (0.73-0.95)
Adjusted OR (95% CI)	1.43 (1.21-1.68)	1.41 (0.98-2.03)	2.21 (1.71-2.84)	2.99 (2.07-4.33)	3.09 (2.58-3.71)	0.73 (0.64-0.83)
**Comparison with private, individually purchased coverage**						
Medicare (n = 66 098)						
Unadjusted OR (95% CI)	0.36 (0.32-0.40)	2.68 (2.23-3.22)	1.37 (1.21-1.55)	0.99 (0.87-1.14)	1.55 (1.41-1.71)	0.68 (0.63-0.74)
Adjusted OR (95% CI)	0.50 (0.44-0.57)	2.56 (2.11-3.12)	1.97 (1.70-2.28)	1.64 (1.40-1.92)	2.34 (2.09-2.63)	0.59 (0.54-0.65)
Medicaid (n = 29 556)						
Unadjusted OR (95% CI)	1.13 (0.99-1.29)	0.57 (0.48-0.67)	0.66 (0.58-0.76)	0.64 (0.55-0.75)	1.28 (1.13-1.46)	1.08 (0.97-1.20)
Adjusted OR (95% CI)	1.50 (1.30-1.72)	0.59 (0.49-0.70)	0.82 (0.71-0.96)	0.88 (0.74-1.06)	1.62 (1.41-1.86)	0.98 (0.87-1.10)
VHA or military (n = 22 896)						
Unadjusted OR (95% CI)	1.07 (0.91-1.26)	2.65 (1.85-3.81)	1.94 (1.52-2.47)	2.52 (1.76-3.62)	2.12 (1.76-2.55)	0.74 (0.65-0.85)
Adjusted OR (95% CI)	1.35 (1.13-1.62)	2.38 (1.65-3.44)	2.17 (1.68-2.80)	3.31 (2.29-4.78)	2.52 (2.07-3.06)	0.72 (0.62-0.83)

Abbreviations: OR, odds ratio; VHA, Veterans Health Administration.

^a^ Questions in the Behavioral Risk Factor Surveillance System survey on access, costs, and satisfaction with care are given in the Methods section. The model was adjusted for number of comorbidities and functional impairments, self-reported health, and number of visits for health care. The reference for each model is the public insurance program (eg, Medicare, Medicaid, or VHA or military). Overall missing data in multivariable models ranged from 6.4% to 7.9%.

### Comparison of Private Insurance and Medicaid

Comparisons between private insurance and Medicaid showed mixed results. Individuals with employer-sponsored insurance were more likely to report having a personal physician (OR, 1.58; 95% CI, 1.40-1.77) and less likely to report instability in insurance coverage (OR, 0.34; 95% CI, 0.29-0.39), difficulty seeing a physician because of costs (OR, 0.83; 95% CI, 0.73-0.95), and not taking medication because of costs (OR, 0.78; 95% CI, 0.66-0.92) compared with those covered by Medicaid. However, individuals with employer-sponsored insurance were more likely to report medical debt compared with individuals covered by Medicaid (OR, 2.06; 95% CI, 1.83-2.32) (Table 2 and eTable 3 in the Supplement).

Individuals with individually purchased insurance were more likely to report having a personal physician (OR, 1.50; 95% CI, 1.30-1.72) and less likely to report instability in insurance coverage (OR, 0.59; 95% CI, 0.49-0.70) and difficulty seeing a physician because of costs (OR, 0.82; 95% CI, 0.71-0.96), but there was no difference in reports of not taking medication because of costs (OR, 0.88; 95% CI, 0.74-1.06) compared with individuals covered by Medicaid. Individuals with individually purchased insurance were more likely to report medical debt compared with individuals covered by Medicaid (OR, 1.62; 95% CI, 1.41-1.86) (Table 2 and eTable 4 in the Supplement).

### Comparison of Private Insurance and VHA or Military Insurance

Individuals with employer-sponsored private insurance were more likely to report having a personal physician compared with those with VHA or military coverage (OR, 1.43; 95% CI, 1.21-1.68). However, individuals covered by employer-sponsored private insurance were also more likely to report instability in insurance coverage (OR, 1.41; 95% CI, 0.98-2.03), difficulty seeing a physician because of costs (OR, 2.21; 95% CI, 1.71-2.84), difficulty taking medications because of costs (OR, 2.99; 95% CI, 2.07-4.33), medical debt (OR, 3.09; 95% CI, 2.58-3.71), and less satisfaction with care (OR, 0.73; 95% CI, 0.64-0.83) compared with individuals who had VHA or military coverage (Table 2 and eTable 5 in the Supplement).

Individuals with individually purchased private insurance were more likely to report having a personal physician compared with those who had VHA or military coverage (OR, 1.35; 95% CI, 1.13-1.62). However, individuals covered by individually purchased private insurance were more likely to report instability in insurance coverage (OR, 2.38; 95% CI, 1.65-3.44), difficulty seeing a physician because of costs (OR, 2.17; 95% CI, 1.68-2.80), difficulty taking medications because of costs (OR, 3.31; 95% CI, 2.29-4.78), medical debt (OR, 2.52; 95% CI, 2.07-3.06), and less satisfaction with care (OR, 0.72; 95% CI, 0.62-0.83) compared with individuals who had VHA or military coverage (Table 2 and eTable 6 in the Supplement).

## Discussion

This survey study found differences in experiences related to access to care, costs of care, and satisfaction with care among public and private health insurance programs in the US. In analyses adjusted for baseline health status, individuals covered by employer-sponsored and individually purchased private insurance were less likely to report having a personal physician, stability in insurance coverage, and satisfaction with care compared with those covered by Medicare. Moreover, individuals with private health insurance were more likely to report difficulty seeing physicians because of cost, not taking medications because of costs, and having medical debt compared with individuals covered by Medicare. Similar patterns were observed in comparisons between private insurance and VHA or military coverage.

These data are consistent with findings from prior research,^14,15,16,26^ provide an update on US adults’ experiences with private and public coverage, and suggest that the experiences of individuals covered by private insurance compare less favorably with the experiences of individuals covered by publicly sponsored plans. Although we did not find recently published research comparing experiences among all 5 forms of coverage, our findings are consistent with data from a 2015 Gallup poll of a national sample of US adults that revealed that individuals with Medicare and VHA or military coverage were the most satisfied with the care they received.^27^ Our findings are also consistent with results of research conducted in 2000 and 2010 that suggested greater overall satisfaction among Medicare beneficiaries compared with those covered by employer-sponsored insurance.^14,15,26^

The comparisons focused on private insurance and Medicaid had mixed findings. The states included in our analyses have Medicaid programs with varying benefits, which may account for some of the differences observed. In some states, Medicaid programs have substantial cost sharing, out-of-pocket costs, and copays. For example, Kentucky requires a copay of $50 per day of inpatient hospitalization, whereas Maine charges $3 per day and Delaware charges nothing.^28^ Such costs may pose a burden for individuals with low income and may explain some of the differences in perceptions we observed. However, research suggests that patients with Medicaid have reduced access to care.^20^ Medicaid expansion has benefited individuals without prior insurance,^1,3,22,29^ but access to specific services remains a challenge.^20,21,22^ Prior studies^20,21,22^ found that Medicaid coverage did not favorably compare with private insurance in terms of access to care for specific services, which may explain some of our findings.

A consistent finding between private and public insurance was the experience of medical debt. Those who had either form of private insurance (employer sponsored or individually purchased) were more likely to report medical debt compared with individuals covered by any form of public insurance. This is not surprising given that health care costs are increasing faster than the median income in most states.^4^ Reform efforts directed at increasing subsidies, reducing cost sharing and deductibles, and eliminating surprise medical billing may be important tactics for reducing medical debt experienced by those covered by private plans.^4,30,31,32,33^

Individuals covered by VHA or military insurance reported more positive experiences related to access to, costs of, and satisfaction with care compared with individuals covered by private insurance, with the exception that those with VHA or military coverage were less likely to report having a personal physician. The latter finding may not be surprising given that some of these individuals may have been active-duty personnel who were frequently reassigned or deployed to new locations, and this frequent change in location may have been associated with reduced access to a regular physician. In addition, the VHA has ties to academic medical centers and hosts resident physicians and other trainees, which may lead to a perception of lack of a consistent primary care practioner among veterans. Although the perceptions of individuals with private insurance were less favorable compared with those with VHA or military insurance, the question in the BRFSS survey that captured VHA and military coverage included those covered by the military, Tricare (a health care program of the US Department of Defense Military Health System, which is privately administered), and the VHA. Therefore, the comparisons were limited because of the heterogenous nature of coverage among individuals included in the VHA and military group.

In addition, the differences among the 5 insurance groups may have been less dependent on the source of insurance coverage (eg, public vs private) than on the quality of benefits being offered by each plan. For instance, Medicare beneficiaries, especially those with Medicare supplemental coverage, have historically been shown to experience relatively low out-of-pocket costs and increased satisfaction.^34^ This suggests that our findings may be less a function of the source of coverage (in this case, a public insurer and a private insurer) than the comprehensiveness of the benefit designs.

### Limitations

This study has limitations. First, the BRFSS is a cross-sectional, state-based telephone survey in which data are collected from selected US states, which limits generalizability. However, the 17 states and the District of Columbia assessed in this analysis included a variety of different populations in terms of sociodemographic characteristics, health status, and rural vs urban residence, and the analysis represents the experiences of more than 61 million individuals with insurance coverage. Second, although we attempted to control for factors that we believed could affect our findings, unadjusted differences in underlying sociodemographic characteristics could have led to uncontrolled confounding. Although age and race/ethnicity may be associated with how individuals experience care, we did not adjust for these factors because it is not possible to make adjustments among nonoverlapping groups. Third, the BRFSS categorizes insurance coverage based on self-report. However, the findings of a previous study^35^ in which survey data were compared with enrollment data suggest that self-report may be a viable method to categorize coverage. Fourth, questions on coverage in the BRFSS survey do not distinguish between fee-for-service care and managed care; therefore, we were unable to make a more detailed evaluation of perceptions and experiences of fee-for-service care vs managed care (eg, Medicare Advantage). Fifth, comorbid conditions and functional status were also self-reported; however, the reliability and validity of BRFSS data on medical conditions have been previously validated.^2^ Sixth, the heterogeneity of coverage within each insurance category in both private and public programs was not uniform and varied in terms of benefits by employer, plan, and state and may have confounded our findings.

## Conclusions

In this survey study, individuals covered by private insurance appeared to experience less access to care, higher costs of care, and decreased satisfaction with care compared with individuals with Medicare or VHA or military coverage. As US policy makers continue to debate health insurance reform, efforts directed at increasing the number of individuals covered by Medicare or improving protections for individuals covered by private insurance against increasing out-of-pocket costs, high deductibles, and surprise billing may be associated with improved experience of and satisfaction with health care.

## Notes

Supplement. **eTable 1.** Comparison Employer Sponsored Health Insurance to Medicare on Access, Cost and Satisfaction With Care After Accounting for Health Status, N = 114 124 **eTable 2.** Comparisons of Individually Purchased Private Insurance to Medicare on Access to Care, Cost of Care and Satisfaction With Care After Accounting for Health Status, N = 66 098 **eTable 3.** Comparison Employer Sponsored Health Insurance to Medicaid on Access, Cost and Satisfaction With Care After Accounting for Health Status, N = 77 582 **eTable 4.** Comparisons of Individually Purchased Private Insurance to Medicaid on Access to Care, Cost of Care and Satisfaction With Care After Accounting for Health Status, N = 29 556 **eTable 5.** Comparison Employer Sponsored Health Insurance to VA/Military on Access, Cost and Satisfaction With Care After Accounting for Health Status, N = 70 992 **eTable 6.** Comparisons of Individually Purchased Private Insurance to VA/Military on Access to Care, Cost of Care and Satisfaction With Care After Accounting for Health Status, N = 22 986

## References

[1] Aliu O, Auger KA, Sun GH, . The effect of pre-Affordable Care Act (ACA) Medicaid eligibility expansion in New York State on access to specialty surgical care. Med Care. 2014;52(9):790-795. doi:10.1097/MLR.0000000000000175 24984209 PMC4262819

[2] Angier HE, Marino M, Springer RJ, Schmidt TD, Huguet N, DeVoe JE. The Affordable Care Act improved health insurance coverage and cardiovascular-related screening rates for cancer survivors seen in community health centers. Cancer. 2020;126(14):3303-3311. doi:10.1002/cncr.32900 32294251 PMC7340351

[3] Breathett K, Allen LA, Helmkamp L, . The Affordable Care Act Medicaid expansion correlated with increased heart transplant listings in African-Americans but not Hispanics or Caucasians. JACC Heart Fail. 2017;5(2):136-147. doi:10.1016/j.jchf.2016.10.013 28109783 PMC5291811

[4] Collins SR, Doty MM, Garber T. Adults ages 50-64 and the Affordable Care Act of 2010. Issue Brief (Commonw Fund). 2010;105:1-20. 21155372

[5] Griffith KN, Bor JH. Changes in health care access, behaviors, and self-reported health among low-income US adults through the fourth year of the Affordable Care Act. Med Care. 2020;58(6):574-578. doi:10.1097/MLR.0000000000001321 32221101 PMC8133296

[6] Han X, Zang Xiong K, Kramer MR, Jemal A. The Affordable Care Act and cancer stage at diagnosis among young adults. J Natl Cancer Inst. 2016;108(9):djw058. doi:10.1093/jnci/djw058 27140956

[7] Hofer AN, Abraham JM, Moscovice I. Expansion of coverage under the Patient Protection and Affordable Care Act and primary care utilization. Milbank Q. 2011;89(1):69-89. doi:10.1111/j.1468-0009.2011.00620.x 21418313 PMC3160595

[8] Hong YR, Holcomb D, Bhandari M, Larkin L. Affordable Care Act: comparison of healthcare indicators among different insurance beneficiaries with new coverage eligibility. BMC Health Serv Res. 2016;16:114. doi:10.1186/s12913-016-1362-1 27044311 PMC4820965

[9] Nguyen BT, Han X, Jemal A, Drope J. Diet quality, risk factors and access to care among low-income uninsured American adults in states expanding Medicaid vs. states not expanding under the affordable care act. Prev Med. 2016;91:169-171. doi:10.1016/j.ypmed.2016.08.015 27514245

[10] Sineshaw HM, Ellis MA, Yabroff KR, . Association of Medicaid expansion under the Affordable Care Act with stage at diagnosis and time to treatment initiation for patients with head and neck squamous cell carcinoma. JAMA Otolaryngol Head Neck Surg. 2020;146(3):247-255. doi:10.1001/jamaoto.2019.4310 31944232 PMC6990851

[11] Toyoda Y, Oh EJ, Premaratne ID, Chiuzan C, Rohde CH. Affordable Care Act state-specific Medicaid expansion: impact on health insurance coverage and breast cancer screening rate. J Am Coll Surg. 2020;S1072-7515(20)30213-1. doi:10.1016/j.jamcollsurg.2020.01.031 32273233

[12] Garfield R, Orgera K, Damico A. The uninsured and the ACA: a primer—key facts about health insurance and the uninsured amidst changes to the Affordable Care Act. Kaiser Family Foundation. Published January 25, 2019. Accessed April 3, 2021. https://www.kff.org/uninsured/report/the-uninsured-and-the-aca-a-primer-key-facts-about-health-insurance-and-the-uninsured-amidst-changes-to-the-affordable-care-act/

[13] Kaiser Family Foundation. Health care and the 2020 presidential election. Published October 6, 2020. Accessed November 11, 2020. https://www.kff.org/slideshow/health-care-and-the-2020-presidential-election/

[14] Hoffman C, Schoen C, Rowland D, Davis K. Gaps in health coverage among working-age Americans and the consequences. J Health Care Poor Underserved. 2001;12(3):272-289. doi:10.1353/hpu.2010.0739 11475546

[15] Davis K, Stremikis K, Doty MM, Zezza MA. Medicare beneficiaries less likely to experience cost- and access-related problems than adults with private coverage. Health Aff (Millwood). 2012;31(8):1866-1875. doi:10.1377/hlthaff.2011.1357 22813985

[16] Catlin MK, Poisal JA, Cowan CA. Out-of-pocket health care expenditures, by insurance status, 2007-10. Health Aff (Millwood). 2015;34(1):111-116. doi:10.1377/hlthaff.2014.0422 25561651

[17] Sarkar M, Earley ER, Asti L, Chisolm DJ. Differences in health care needs, health care utilization, and health care outcomes among children with special health care needs in Ohio: a comparative analysis between Medicaid and private insurance. J Public Health Manag Pract. 2017;23(1):e1-e9. doi:10.1097/PHH.0000000000000403 27870721

[18] Mahal AR, Mahal BA, Nguyen PL, Yu JB. Prostate cancer outcomes for men aged younger than 65 years with Medicaid versus private insurance. Cancer. 2018;124(4):752-759. doi:10.1002/cncr.31106 29084350

[19] Parsons HM, Maguire FB, Morris CR, . Impact of insurance type and timing of Medicaid enrollment on survival among adolescents and young adults with cancer. Pediatr Blood Cancer. 2020;67(9):e28498. doi:10.1002/pbc.28498 32589358

[20] Hsiang WR, Lukasiewicz A, Gentry M, . Medicaid patients have greater difficulty scheduling health care appointments compared with private insurance patients: a meta-analysis. Inquiry. 2019;56:46958019838118. doi:10.1177/0046958019838118 30947608 PMC6452575

[21] Lee YH, Chen AX, Varadaraj V, . Comparison of access to eye care appointments between patients with Medicaid and those with private health care insurance. JAMA Ophthalmol. 2018;136(6):622-629. doi:10.1001/jamaophthalmol.2018.0813 29710290 PMC6007883

[22] Chaiyachati KH, Hom JK, Wong C, . Access to primary and dental care among adults newly enrolled in Medicaid. Am J Manag Care. 2019;25(3):135-139. 30875182

[23] US Centers for Disease Control and Prevention. The Behavioral Risk Factor Surveillance System (BRFSS). August 31, 2020. Accessed November 11, 2020. https://www.cdc.gov/brfss/index.html

[24] Schoen C, Davis K, Willink A. Medicare beneficiaries’ high out-of-pocket costs: cost burdens by income and health status. Issue Brief (Commonw Fund). 2017;11:1-14. 28498650

[25] Xiao H, Barber JP. The effect of perceived health status on patient satisfaction. Value Health. 2008;11(4):719-725. doi:10.1111/j.1524-4733.2007.00294.x 18179667

[26] Doty MM, Schoen C, Davis K. Medicare vs. private insurance: rhetoric and reality. *Health Affairs*. Published October 9, 2002. Accessed April 3, 2021. https://www.commonwealthfund.org/publications/journal-article/2002/oct/medicare-vs-private-insurance-rhetoric-and-reality 10.1377/hlthaff.w2.311 12703587

[27] Riffkin R. Americans with government health plans most satisfied. Published November 6, 2015. Accessed January 6, 2021. https://news.gallup.com/poll/186527/americans-government-health-plans-satisfied.aspx

[28] Kaiser Family Foundation. Medicaid & CHIP indicators: premium and cost-sharing requirements. Accessed November 11, 2020. https://www.kff.org/state-category/medicaid-chip/premium-cost-sharing-requirements/

[29] Moniz MH, Kirch MA, Solway E, . Association of access to family planning services with Medicaid expansion among female enrollees in Michigan. JAMA Netw Open. 2018;1(4):e181627. doi:10.1001/jamanetworkopen.2018.1627 30646135 PMC6324283

[30] Busch SH, Kyanko KA. Incorrect provider directories associated with out-of-network mental health care and outpatient surprise bills. Health Aff (Millwood). 2020;39(6):975-983. doi:10.1377/hlthaff.2019.01501 32479225 PMC7497897

[31] Chhabra KR, Dimick JB. “Surprise” out-of-network medical bills. JAMA. 2020;323(9):902. doi:10.1001/jama.2020.0847 32125405

[32] Chhabra KR, Sheetz KH, Nuliyalu U, Dekhne MS, Ryan AM, Dimick JB. Out-of-network bills for privately insured patients undergoing elective surgery with in-network primary surgeons and facilities. JAMA. 2020;323(6):538-547. doi:10.1001/jama.2019.21463 32044941 PMC7042888

[33] Cooper Z, Scott Morton F. Out-of-network emergency-physician bills—an unwelcome surprise. N Engl J Med. 2016;375(20):1915-1918. doi:10.1056/NEJMp1608571 27959612

[34] Park S, White L, Fishman P, Larson EB, Coe NB. Health care utilization, care satisfaction, and health status for Medicare Advantage and traditional Medicare beneficiaries with and without Alzheimer disease and related dementias. JAMA Netw Open. 2020;3(3):e201809. doi:10.1001/jamanetworkopen.2020.1809 32227181 PMC7485599

[35] Pascale J, Fertig AR, Call KT. Assessing the accuracy of survey reports of health insurance coverage using enrollment data. Health Serv Res. 2019;54(5):1099-1109. doi:10.1111/1475-6773.13191 31287571 PMC6736923

