# Supplementary material for: Access to Care, Cost of Care, and Satisfaction With Care Among Adults With Private and Public Health Insurance in the US
Source: JAMA Netw Open. 2021 Jun 1;4(6):e2110275. doi: 10.1001/jamanetworkopen.2021.10275 (PMC8170543; doi:10.1001/jamanetworkopen.2021.10275)
Supplement: Supplement. — eTable 1. Comparison Employer Sponsored Health Insurance to Medicare on Access, Cost and Satisfaction With Care After Accounting for Health Status, N = 114 124 eTable 2. Comparisons of Individually Purchased Private Insurance to Medicare on Access to Care, Cost of Care and Satisfaction With Care After Accounting for Health Status, N = 66 098 eTable 3. Comparison Employer Sponsored Health Insurance to Medicaid on Access, Cost and Satisfaction With Care After Accounting for Health Status, N = 77 582 eTable 4. Comparisons of Individually Purchased Private Insurance to Medicaid on Access to Care, Cost of Care and Satisfaction With Care After Accounting for Health Status, N = 29 556 eTable 5. Comparison Employer Sponsored Health Insurance to VA/Military on Access, Cost and Satisfaction With Care After Accounting for Health Status, N = 70 992 eTable 6. Comparisons of Individually Purchased Private Insurance to VA/Military on Access to Care, Cost of Care and Satisfaction With Care After Accounting for Health Status, N = 22 986 [file jamanetwopen-e2110275-s001.pdf]

Supplementary Online Content

Wray CM, Khare M, Keyhani S. Access to care, cost of care, and satisfaction with care among adults with private and public health insurance in the US. *JAMA Netw Open*. 2021;4(6):e2110275. doi:10.1001/jamanetworkopen.2021.10275

eTable 1. Comparison Employer Sponsored Health Insurance to Medicare on Access, Cost and Satisfaction With Care After Accounting for Health Status, N = 114 124
eTable 2. Comparisons of Individually Purchased Private Insurance to Medicare on Access to Care, Cost of Care and Satisfaction With Care After Accounting for Health Status, N = 66 098
eTable 3. Comparison Employer Sponsored Health Insurance to Medicaid on Access, Cost and Satisfaction With Care After Accounting for Health Status, N = 77 582
eTable 4. Comparisons of Individually Purchased Private Insurance to Medicaid on Access to Care, Cost of Care and Satisfaction With Care After Accounting for Health Status, N = 29 556
eTable 5. Comparison Employer Sponsored Health Insurance to VA/Military on Access, Cost and Satisfaction With Care After Accounting for Health Status, N = 70 992
eTable 6. Comparisons of Individually Purchased Private Insurance to VA/Military on Access to Care, Cost of Care and Satisfaction With Care After Accounting for Health Status, N = 22 986

This supplementary material has been provided by the authors to give readers additional information about their work.

© 2021 Wray CM et al. *JAMA Network Open*.

eTable 1. Comparison Employer Sponsored Health Insurance to Medicare on Access, Cost and Satisfaction With Care After Accounting for Health Status*, N = 114 124

	**Access to Care**		**Costs of Care**			**Satisfaction with Care**
	**Has a personal doctor**	**Instability in insurance coverage**	**Difficulty seeing a doctor due to cost**	**Not taking medications due to costs**	**Reported medical debt**	**Very satisfied with care*** **
N**	113,852	113,996	113,942	107,059	113,510	112,144
	Unadjusted OR (95% CI)					
	Adjusted OR (95% CI) **					
Employer Sponsored Insurance Compared to Medicare	0.38(0.35,0.41)<br>0.52(0.48,0.57)	1.38(1.19,1.6)<br>1.54(1.3,1.83)	1.04(0.95,1.14)<br>2.00(1.77,2.27)	0.66 (0.60,0.73)<br>1.44(1.27,1.62)	1.65(1.55,1.76)<br>2.92(2.69,3.17)	0.76(0.72,0.81)<br>0.60(0.56,0.64)
	Adjusted OR (95% CI) **					
Self-Reported Health						
Excellent/Very good/Good (reference)						
Fair/poor	0.58(0.54,0.64)	0.58(0.53,0.64)	0.56(0.52,0.62)	0.59(0.54,0.64)	0.53(0.49,0.58)	0.55(0.50,0.60)
Number of Visits to the Doctor, Nurse, or Other Health Professional in Past 12 Months						
None (reference)						
1-2	0.79(0.70,0.90)	0.67(0.59,0.76)	0.68(0.6,0.77)	0.65(0.56,0.75)	0.65(0.57,0.74)	0.72(0.63,0.83)
>2	0.66(0.58,0.75)	0.54(0.48,0.61)	0.54(0.48,0.61)	0.53(0.46,0.61)	0.49(0.43,0.55)	0.58(0.51,0.66)
Number of Comorbid Conditions						
None (reference)						
1	0.64(0.61,0.69)	0.63(0.59,0.67)	0.63(0.59,0.67)	0.61(0.58,0.66)	0.62(0.58,0.66)	0.63(0.59,0.67)
2	0.36(0.33,0.4)	0.35(0.32,0.38)	0.35(0.32,0.38)	0.34(0.31,0.38)	0.34(0.31,0.37)	0.35(0.32,0.38)
>2	0.26(0.23,0.3)	0.25(0.22,0.28)	0.25(0.22,0.29)	0.24(0.21,0.28)	0.24(0.21,0.27)	0.25(0.22,0.29)
Number of Functional Impairments						
None (reference)						
1	0.39(0.36,0.43)	0.39(0.36,0.42)	0.37(0.34,0.41)	0.39(0.35,0.42)	0.36(0.33,0.4)	0.38(0.34,0.41)
>1	0.24(0.22,0.28)	0.24(0.21,0.27)	0.22(0.19,0.24)	0.23(0.21,0.26)	0.21(0.18,0.23)	0.23(0.20,0.25)

* Questions posed in BRFSS on access, costs and satisfaction with care:
-Do you have one person you think of as your personal doctor or health care provider?
-In the past 12 months is there any time when you did not have any health insurance or coverage? (instability in insurance coverage)
-Was there a time in the past 12 months when you needed to see a doctor but could not because of cost? (difficulty seeing a doctor due to cost)
-In the past 12 months, was there a time when you did not take your prescription medications due to cost? (not taking Medication due to cost)
-Do you currently have any health care bills that are being paid off over time? (medical debt)
-In general, how satisfied are you with the care you received? Response categorization: Very satisfied versus somewhat satisfied/not satisfied (Very satisfied with care)

**Adjusted for number of comorbid conditions, number of functional impairments and number of visits. Missing data was dropped from the analyses.

© 2021 Wray CM et al. *JAMA Network Open*.

eTable 2. Comparisons of Individually Purchased Private Insurance to Medicare on Access to Care, Cost of Care and Satisfaction With Care After Accounting for Health Status*, N = 66 098

	**Access to Care**		**Costs of Care**			**Satisfaction with Care**
	**Has a personal doctor**	**Instability in insurance coverage**	**Difficulty seeing a doctor due to cost**	**Not taking medications due to costs**	**Reported medical debt**	**Very satisfied with care*** **
N**	65,882	66,004	65,965	62,883	65,647	64,887
	Unadjusted OR (95% CI)					
	Adjusted OR (95% CI)					
Individually Purchased Private Insurance Compared to Medicare	0.36(0.32,0.40)<br>0.50(0.44,0.57)	2.68(2.23,3.22)<br>2.56(2.11,3.12)	1.37(1.21,1.55)<br>1.97(1.70,2.28)	0.99(0.87,1.14)<br>1.64(1.40,1.92)	1.55(1.41,1.71)<br>2.34(2.09,2.63)	0.68(0.63,0.74)<br>0.59(0.54,0.65)
	Adjusted OR (95% CI)**					
Self-Reported Health						
Excellent/Very good/Good (reference)						
Fair/poor	0.77(0.69,0.86)	0.77(0.69,0.87)	0.75(0.67,0.84)	0.76(0.68,0.85)	0.74(0.65,0.83)	0.73(0.65,0.82)
Number of Visits to the Doctor, Nurse, or Other Health Professional in Past 12 Months						
None (reference)						
1-2	0.75(0.63,0.89)	0.62(0.52,0.73)	0.62(0.52,0.73)	0.61(0.51,0.74)	0.59(0.50,0.70)	0.69(0.58,0.83)
>2	0.61(0.52,0.73)	0.50(0.42,0.58)	0.48(0.41,0.57)	0.49(0.41,0.58)	0.44(0.37,0.52)	0.55(0.46,0.65)
Number of Comorbid Conditions						
None (reference)						
1	0.57(0.52,0.63)	0.56(0.51,0.61)	0.55(0.50,0.60)	0.55(0.50,0.60)	0.55(0.51,0.61)	0.55(0.50,0.61)
2	0.43(0.38,0.49)	0.42(0.37,0.48)	0.41(0.36,0.47)	0.41(0.36,0.46)	0.40(0.35,0.46)	0.42(0.37,0.48)
>2	0.32(0.27,0.38)	0.31(0.26,0.37)	0.30(0.25,0.36)	0.29(0.25,0.35)	0.29(0.24,0.35)	0.30(0.25,0.36)
Number of Functional Impairments						
None (reference)						
1	0.55(0.49,0.62)	0.54(0.48,0.6)	0.53(0.47,0.59)	0.53(0.48,0.6)	0.52(0.46,0.58)	0.53(0.48,0.60)
>1	0.46(0.40,0.54)	0.44(0.37,0.52)	0.41(0.35,0.48)	0.45(0.38,0.53)	0.40(0.34,0.47)	0.43(0.37,0.51)

* Questions posed in BRFSS on access, costs and satisfaction with care:
-Do you have one person you think of as your personal doctor or health care provider?
-In the past 12 months is there any time when you did not have any health insurance or coverage? (instability in insurance coverage)
-Was there a time in the past 12 months when you needed to see a doctor but could not because of cost? (difficulty seeing a doctor due to cost)
-In the past 12 months, was there a time when you did not take your prescription medications due to cost? (not taking Medication due to cost)
-Do you currently have any health care bills that are being paid off over time? (medical debt)
-In general, how satisfied are you with the care you received? Response categorization: Very satisfied versus somewhat satisfied/not satisfied (Very satisfied with care)

**Adjusted for number of comorbid conditions, number of functional impairments and number of visits.

© 2021 Wray CM et al. *JAMA Network Open*.

eTable 3. Comparison Employer Sponsored Health Insurance to Medicaid on Access, Cost and Satisfaction With Care After Accounting for Health Status*, N = 77 582

	Access to Care		Costs of Care			Satisfaction with Care
	**Has a personal doctor**	**Instability in insurance coverage**	**Difficulty seeing a doctor due to cost**	**Not taking medications due to costs**	**Reported medical debt**	**Very satisfied with care*** **
N**	77,408	77,464	77,460	71,567	77,114	76,138
	Unadjusted OR (95% CI)					
	Adjusted OR (95% CI) **					
Employer Sponsored Insurance Compared to Medicaid	1.19(1.08,1.32)<br>1.58(1.40,1.77)	0.29(0.26,0.33)<br>0.34(0.29,0.39)	0.50(0.45,0.56)<br>0.83(0.73,0.95)	0.43(0.37,0.49)<br>0.78(0.66,0.92)	1.36(1.23,1.51)<br>2.06(1.83,2.32)	1.21(1.11,1.32)<br>0.96(0.87,1.06)
	Adjusted OR (95% CI)**					
Self-Reported Health						
Excellent/Very good/Good (reference)						
Fair/poor	0.42(0.37,0.48)	0.42(0.38,0.48)	0.42(0.38,0.48)	0.43(0.38,0.48)	0.40(0.35,0.45)	0.41(0.37,0.47)
Number of Visits to the Doctor, Nurse, or Other Health Professional in Past 12 Months						
None (reference)						
1-2	1.06(0.89,1.27)	1.12(0.93,1.35)	1.20(1.00,1.44)	1.22(0.98,1.51)	1.14(0.95,1.36)	1.21(0.99,1.46)
>2	0.79(0.66,0.94)	0.85(0.7,1.02)	0.94(0.79,1.11)	0.95(0.78,1.17)	0.84(0.71,1)	0.95(0.79,1.14)
Number of Comorbid Conditions						
None (reference)						
1	0.98(0.88,1.09)	0.99(0.88,1.1)	1.00(0.89,1.11)	1(0.89,1.12)	0.98(0.88,1.09)	1.0(0.90,1.11)
2	0.96(0.83,1.12)	0.97(0.83,1.13)	1.00(0.86,1.16)	1.01(0.87,1.18)	0.99(0.85,1.15)	1.0(0.86,1.17)
>2	0.83(0.68,1.01)	0.84(0.68,1.03)	0.87(0.71,1.06)	0.89(0.73,1.09)	0.87(0.72,1.06)	0.9(0.74,1.10)
Number of Functional Impairments						
None (reference)						
1	0.41(0.36,0.46)	0.43(0.38,0.49)	0.42(0.37,0.48)	0.42(0.37,0.47)	0.39(0.34,0.45)	0.41(0.36,0.46)
>1	0.19(0.16,0.22)	0.2(0.17,0.24)	0.20(0.17,0.23)	0.20(0.17,0.23)	0.18(0.15,0.21)	0.19(0.17,0.22)

* Questions posed in BRFSS on access, costs and satisfaction with care:
-Do you have one person you think of as your personal doctor or health care provider?
-In the past 12 months is there any time when you did not have any health insurance or coverage? (instability in insurance coverage)
-Was there a time in the past 12 months when you needed to see a doctor but could not because of cost? (difficulty seeing a doctor due to cost)
-In the past 12 months, was there a time when you did not take your prescription medications due to cost? (not taking Medication due to cost)
-Do you currently have any health care bills that are being paid off over time? (medical debt)
-In general, how satisfied are you with the care you received? Response categorization: Very satisfied versus somewhat satisfied/not satisfied (Very satisfied with care)

**Adjusted for number of comorbid conditions, number of functional impairments and number of visits. Missing data was dropped from the analyses.

© 2021 Wray CM et al. *JAMA Network Open*.

eTable 4. Comparisons of Individually Purchased Private Insurance to Medicaid on Access to Care, Cost of Care and Satisfaction With Care After Accounting for Health Status*, N = 29 556

	**Access to Care**		**Costs of Care**			**Satisfaction with Care**
	**Has a personal doctor**	**Instability in insurance coverage**	**Difficulty seeing a doctor due to cost**	**Not taking medications due to costs**	**Reported medical debt**	**Very satisfied with care*** **
n	29,438	29,472	29,483	27,391	29,251	28,881
	Unadjusted OR (95% CI)					
	Adjusted OR (95% CI) **					
Individually Purchased Private Insurance Compared to Medicaid	1.13(0.99,1.29)<br>1.50(1.30,1.72)	0.57(0.48,0.67)<br>0.59(0.49,0.70)	0.66(0.58,0.76)<br>0.82(0.71,0.96)	0.64(0.55,0.75)<br>0.88(0.74,1.06)	1.28(1.13,1.46)<br>1.62(1.41,1.86)	1.08(0.97,1.20)<br>0.98(0.87,1.10)
	Adjusted OR (95% CI)**					
Self-Reported Health						
Excellent/Very good/Good (reference)						
Fair/poor	0.56(0.48,0.64)	0.55(0.48,0.63)	0.57(0.49,0.65)	0.55(0.48,0.64)	0.55(0.48,0.64)	0.55(0.47,0.63)
Number of Visits to the Doctor, Nurse, or Other Health Professional in Past 12 Months						
None (reference)						
1-2	0.97(0.78,1.19)	1.05(0.84,1.31)	1.10(0.89,1.35)	1.17(0.92,1.48)	1.04(0.84,1.28)	1.16(0.93,1.44)
>2	0.72(0.59,0.89)	0.80(0.65,0.99)	0.84(0.68,1.03)	0.89(0.7,1.11)	0.76(0.62,0.94)	0.89(0.72,1.10)
Number of Comorbid Conditions						
None (reference)						
1	0.84(0.74,0.95)	0.85(0.75,0.97)	0.87(0.76,0.98)	0.87(0.77,1)	0.86(0.76,0.98)	0.87(0.77,0.99)
2	1.15(0.96,1.38)	1.16(0.97,1.4)	1.20(1.00,1.44)	1.2(1,1.45)	1.17(0.98,1.40)	1.20(1.00,1.44)
>2	1.01(0.8,1.28)	1.03(0.82,1.3)	1.06(0.84,1.34)	1.07(0.85,1.36)	1.07(0.85,1.35)	1.10(0.87,1.39)
Number of Functional Impairments						
None (reference)						
1	0.55(0.48,0.64)	0.58(0.5,0.67)	0.56(0.49,0.65)	0.57(0.49,0.66)	0.55(0.48,0.64)	0.56(0.49,0.65)
>1	0.34(0.29,0.41)	0.36(0.3,0.43)	0.35(0.29,0.42)	0.36(0.3,0.44)	0.33(0.28,0.40)	0.35(0.29,0.42)

* Questions posed in BRFSS on access, costs and satisfaction with care:
-Do you have one person you think of as your personal doctor or health care provider?
-In the past 12 months is there any time when you did not have any health insurance or coverage? (instability in insurance coverage)
-Was there a time in the past 12 months when you needed to see a doctor but could not because of cost? (difficulty seeing a doctor due to cost)
-In the past 12 months, was there a time when you did not take your prescription medications due to cost? (not taking Medication due to cost)
-Do you currently have any health care bills that are being paid off over time? (medical debt)
-In general, how satisfied are you with the care you received? Response categorization: Very satisfied versus somewhat satisfied/not satisfied (Very satisfied with care)

**Adjusted for number of comorbid conditions, number of functional impairments and number of visits. Missing data was dropped from the analyses.

© 2021 Wray CM et al. *JAMA Network Open*.

eTable 5. Comparison Employer Sponsored Health Insurance to VA/Military on Access, Cost and Satisfaction With Care After Accounting for Health Status*, N = 70 992

	Access to Care		Costs of Care			Satisfaction with Care
	**Has a personal doctor**	**Instability in insurance coverage**	**Difficulty seeing a doctor due to cost**	**Not taking medications due to costs**	**Reported medical debt**	**Very satisfied with care*** **
N**	70,784	70,853	70,839	65,334	70,577	69,652
	Unadjusted OR (95% CI)					
	Adjusted OR (95% CI) **					
Employer Sponsored Health Insurance to VA/Military	1.13(0.97,1.3)<br>1.43(1.21,1.68)	1.37(0.97,1.93)<br>1.41(0.98,2.03)	1.48(1.17,1.86)<br>2.21(1.71,2.84)	1.68(1.19,2.38)<br>2.99(2.07,4.33)	2.25(1.9,2.67)<br>3.09(2.58,3.71)	0.83(0.73,0.95)<br>0.73(0.64,0.83)
	Adjusted OR (95% CI)**					
Self-Reported Health						
Excellent/Very good/Good (reference)						
Fair/poor	0.75(0.63,0.9)	0.74(0.62,0.88)	0.72(0.60,0.86)	0.76(0.63,0.9)	0.69(0.57,0.83)	0.71(0.59,0.85)
Number of Visits to the Doctor, Nurse, or Other Health Professional in Past 12 Months						
None (reference)						
1-2	0.64(0.49,0.84)	0.69(0.53,0.9)	0.72(0.55,0.93)	0.75(0.57,0.98)	0.69(0.53,0.89)	0.69(0.53,0.89)
>2	0.65(0.50,0.84)	0.72(0.57,0.92)	0.74(0.58,0.95)	0.79(0.61,1.02)	0.67(0.52,0.85)	0.67(0.52,0.85)
Number of Comorbid Conditions						
None (reference)						
1	0.98(0.86,1.13)	1(0.87,1.14)	0.99(0.87,1.14)	0.93(0.81,1.07)	0.98(0.86,1.13)	0.99(0.86,1.14)
2	0.66(0.56,0.79)	0.69(0.58,0.83)	0.69(0.58,0.82)	0.64(0.54,0.77)	0.67(0.56,0.8)	0.70(0.58,0.83)
>2	0.36(0.28,0.46)	0.38(0.29,0.49)	0.39(0.30,0.50)	0.34(0.26,0.43)	0.36(0.28,0.47)	0.39(0.30,0.50)
Number of Functional Impairments						
None (reference)						
1	0.52(0.43,0.63)	0.51(0.42,0.62)	0.49(0.40,0.59)	0.48(0.4,0.58)	0.48(0.4,0.58)	0.50(0.41,0.60)
>1	0.29(0.24,0.36)	0.29(0.24,0.36)	0.26(0.21,0.32)	0.25(0.2,0.31)	0.25(0.21,0.32)	0.28(0.23,0.35)

* Questions posed in BRFSS on access, costs and satisfaction with care:
-Do you have one person you think of as your personal doctor or health care provider?
-In the past 12 months is there any time when you did not have any health insurance or coverage? (instability in insurance coverage)
-Was there a time in the past 12 months when you needed to see a doctor but could not because of cost? (difficulty seeing a doctor due to cost)
-In the past 12 months, was there a time when you did not take your prescription medications due to cost? (not taking Medication due to cost)
-Do you currently have any health care bills that are being paid off over time? (medical debt)
-In general, how satisfied are you with the care you received? Response categorization: Very satisfied versus somewhat satisfied/not satisfied (Very satisfied with care)

**Adjusted for number of comorbid conditions, number of functional impairments and number of visits. Missing data was dropped from the analyses.

© 2021 Wray CM et al. *JAMA Network Open*.

eTable 6. Comparisons of Individually purchased Private Insurance to VA/Military on Access to Care, Cost of Care and Satisfaction With Care After Accounting for Health Status*, N = 22 986

	**Access to Care**		**Costs of Care**			**Satisfaction with Care**
	**Has a personal doctor**	**Instability in insurance coverage**	**Difficulty seeing a doctor due to cost**	**Not taking medications due to costs**	**Reported medical debt**	**Very satisfied with care*** **
N**	22,814	22,861	22,862	21,158	22,714	22,395
	Unadjusted OR (95% CI)					
	Adjusted OR (95% CI) **					
Individually Purchased Private Insurance Compared to VA/Military	1.07(0.91,1.26)<br>1.35(1.13,1.62)	2.65(1.85,3.81)<br>2.38(1.65,3.44)	1.94(1.52,2.47)<br>2.17(1.68,2.80)	2.52(1.76,3.62)<br>3.31(2.29,4.78)	2.12(1.76,2.55)<br>2.52(2.07,3.06)	0.74(0.65,0.85)<br>0.72(0.62,0.83)
	Adjusted OR (95% CI)**					
Self-Reported Health						
Excellent/Very good/Good (reference)						
Fair/poor	1(0.82,1.21)	0.99(0.82,1.2)	0.97(0.8,1.18)	1.00(0.82,1.21)	0.96(0.79,1.17)	0.95(0.78,1.16)
Number of Visits to the Doctor, Nurse, or Other Health Professional in Past 12 Months						
None (reference)						
1-2	0.58(0.43,0.78)	0.63(0.48,0.84)	0.65(0.49,0.86)	0.7(0.52,0.94)	0.62(0.47,0.82)	0.68(0.52,0.88)
>2	0.58(0.44,0.77)	0.65(0.50,0.85)	0.65(0.50,0.85)	0.7(0.53,0.93)	0.59(0.45,0.77)	0.7(0.54,0.89)
Number of Comorbid Conditions						
None (reference)						
1	0.85(0.73,0.99)	0.87(0.75,1.01)	0.86(0.74,1.01)	0.82(0.7,0.96)	0.86(0.74,1.01)	0.87(0.74,1.02)
2	0.78(0.64,0.96)	0.82(0.67,1)	0.81(0.66,0.99)	0.75(0.61,0.92)	0.77(0.63,0.95)	0.82(0.67,1.01)
>2	0.43(0.33,0.57)	0.47(0.35,0.62)	0.47(0.36,0.63)	0.4(0.3,0.54)	0.44(0.33,0.58)	0.46(0.35,0.61)
Number of Functional Impairments						
None (reference)						
1	0.73(0.6,0.89)	0.72(0.59,0.88)	0.7(0.57,0.86)	0.67(0.55,0.82)	0.71(0.58,0.87)	0.72(0.58,0.88)
>1	0.55(0.44,0.69)	0.55(0.44,0.7)	0.5(0.4,0.63)	0.5(0.39,0.63)	0.5(0.4,0.64)	0.54(0.43,0.68)

* Questions posed in BRFSS on access, costs and satisfaction with care:
-Do you have one person you think of as your personal doctor or health care provider?
-In the past 12 months is there any time when you did not have any health insurance or coverage? (instability in insurance coverage)
-Was there a time in the past 12 months when you needed to see a doctor but could not because of cost? (difficulty seeing a doctor due to cost)
-In the past 12 months, was there a time when you did not take your prescription medications due to cost? (not taking Medication due to cost)
-Do you currently have any health care bills that are being paid off over time? (medical debt)
-In general, how satisfied are you with the care you received? Response categorization: Very satisfied versus somewhat satisfied/not satisfied (Very satisfied with care)

**Adjusted for number of comorbid conditions, number of functional impairments and number of visits. Missing data was dropped from the analyses.

© 2021 Wray CM et al. *JAMA Network Open*.
